# A Prospective Randomized Double Blind Placebo Controlled Trial on the Efficacy of Ethanol Celiac Plexus Neurolysis in Patients with Operable Pancreatic and Periampullary Adenocarcinoma

**DOI:** 10.1016/j.jamcollsurg.2014.12.013

**Published:** 2014-12-17

**Authors:** Harish Lavu, Harry B Lengel, Naomi M Sell, Joseph A Baiocco, Eugene P Kennedy, Theresa P Yeo, Sherry A Burrell, Jordan M Winter, Sarah Hegarty, Benjamin E. Leiby, Charles J Yeo

**Affiliations:** ˆ Department of Surgery and Division of Biostatistics, Thomas Jefferson University, Jefferson Pancreas Biliary and Related Cancer Center, Philadelphia, PA; * School of Nursing, Rutgers University, The State University of New Jersey, Camden, NJ

## Abstract

**Background:**

Ethanol celiac plexus neurolysis (ECPN) has been shown to be effective in reducing cancer-related pain in patients with locally advanced pancreatic and periampullary adenocarcinoma (PPA). This study examined its efficacy in patients undergoing PPA resection.

**Study Design:**

485 patients participated in this prospective, randomized, double blind placebo controlled trial. Patients were stratified by preoperative pain and disease resectability. They received either ECPN (50% ethanol) or 0.9% normal saline placebo control. The primary endpoint was short and long-term pain and secondary endpoints included postoperative morbidity, QOL and overall survival.

**Results:**

Data from 467 patients were analyzed. The primary endpoint, the percentage of PPA patients experiencing a worsening of pain compared to preoperative baseline for resectable patients, was not different between the ethanol and saline groups in either the resectable/pain stratum (22% vs 18%, RR 1.23 (0.34, 4.46)), or the resectable/no pain stratum (37% vs 34%, RR 1.10 (0.67, 1.81)). On multivariable analysis of resected pancreatic ductal adenocarcinoma (PDA) patients, there was a significant reduction in pain in the resectable/pain group, suggesting that surgical resection of the malignancy alone (independent of ECPN) decrements pain to a significant degree.

**Conclusions:**

In this study, we have demonstrated a significant reduction in pain following surgical resection of PPA. However the addition of ECPN did not synergize to result in a further reduction in pain, and in fact its effect may have been masked by surgical resection. Given this, we cannot recommend the use of ECPN to mitigate cancer related pain in resectable PPA patients.

## Introduction

Pancreatic ductal adenocarcinoma (PDA) is the 4^th^ leading cause of cancer death in the United States with an expected 46,420 new cases and 39,590 deaths in 2014.^1^ Surgical resection is the only potentially curative therapy. ^2^ Unfortunately, at the time of diagnosis, the majority of patients are ineligible for tumor resection primarily due to the presence of locally advanced disease, distant metastasis, or significant medical comorbidities precluding surgery. ^3-6^ The five-year survival rate for all patients with PDA is 6% and improves to 15-25% in patients who undergo surgical resection. ^5,7-11^ The treatment strategies employed for PDA are similar to those for ampullary adenocarcinoma, distal cholangiocarcinoma, and duodenal adenocarcinoma, which are the other major cancers that occur within the periampullary region. Taken together, pancreatic and periampullary adenocarcinoma (PPA) presents significant clinical challenges for achieving long-term survival in afflicted patients, and therefore adjunctive and palliative therapies are extremely important in alleviating patient suffering.

Abdominal and back pain are among the most common presenting symptoms in patients with PPA, estimated to affect 30-40% of patients at the time of diagnosis. ^12^ Even in those patients who initially do not present with pain, the majority will ultimately develop this symptom during the course of their disease. ^13,14^ PPA-associated pain is typically unremitting, located in the epigastrium, and can intensify as the disease progresses. ^13,15^ Other symptoms that are associated with and known to cluster with this type of pain include fatigue, insomnia, nausea, diarrhea, weight loss, anxiety, and depression.^16,17^ These symptoms have been documented to have a significant negative impact upon patient quality of life (QOL).^4,17,18^ Current recommendations suggest that the most effective approach to cancer related pain treatment involves using systemic medications titrated in a progressive manner, starting with non-opioid analgesics, moving to weak opioids and then to strong opioids depending upon pain intensity.^19,20^ While opioids can effectively achieve pain relief, they are associated with many adverse side effects. Therefore, nonpharmacological adjuncts, such as ethanol celiac plexus neurolysis (ECPN) have been employed in order to offer effective pain relief while minimizing drug-related side effects.

Despite the first description of celiac plexus neurolysis by Kappis in 1914, clear and convincing evidence supporting the routine use of ECPN in the management of PPA pain is lacking. ^21,22^ The most complete study evaluating this topic was published by Lillemoe et al. in 1993.^23^ This study investigated the efficacy of ECPN in PPA patients found to be unresectable during surgical exploration, demonstrating a significant reduction in pain and an improvement in survival in a small subset of patients with preoperative pain. Subsequent studies have also suggested an improvement in pain in patients with unresectable PPA who have undergone ECPN.^6,24-31^ Despite this strong evidence supporting the use of ECPN in patients with unresectable PPA, to date no studies have evaluated the role of ECPN in patients with resectable PPA. The question remains whether ECPN can be equally effective in reducing PPA- associated pain after surgical resection, and if this will result in an improvement in patient QOL. There is also the question of the theoretical anti-tumor effect of ablating nerves which are infiltrated by malignant cells, which could be hypothesized to exert an influence on cancer recurrence rates and overall survival.

In this trial, we sought to test the hypothesis that intraoperative ECPN would be beneficial for patients with resectable PPA. The primary objective was to evaluate whether ECPN would impact short and long-term tumor-related pain, while secondary endpoints included perioperative complications, QOL and overall survival.

## Methods

### Trial design

We performed a single center, prospective, randomized, double blind placebo controlled trial to evaluate the role of ECPN in mitigating cancer-associated pain in patients with resectable PPA. The trial was approved by both the Jefferson Clinical Cancer Research Review Committee and the Institutional Review Board and is registered with ClinicalTrials.gov (NCT00806611). From December 2008 to August 2013, patients undergoing abdominal exploration for presumed periampullary malignancy at the Thomas Jefferson University Hospital (TJUH) were offered participation in the study. All patients over 18 years requiring major abdominal surgery for PPA were eligible for enrollment with the exclusion of patients not found to have adenocarcinoma on the final pathology and those who had received a previous, preoperative celiac plexus nerve block. At the initial outpatient office visit and after education about the study, informed consent was obtained and patients filled out surveys of preoperative baseline pain (Brief Pain Inventory (BPI)) and QOL (Functional Assessment of Cancer Therapy - Hepatobiliary (FACT-Hep), version 4).

**Figure 1:**
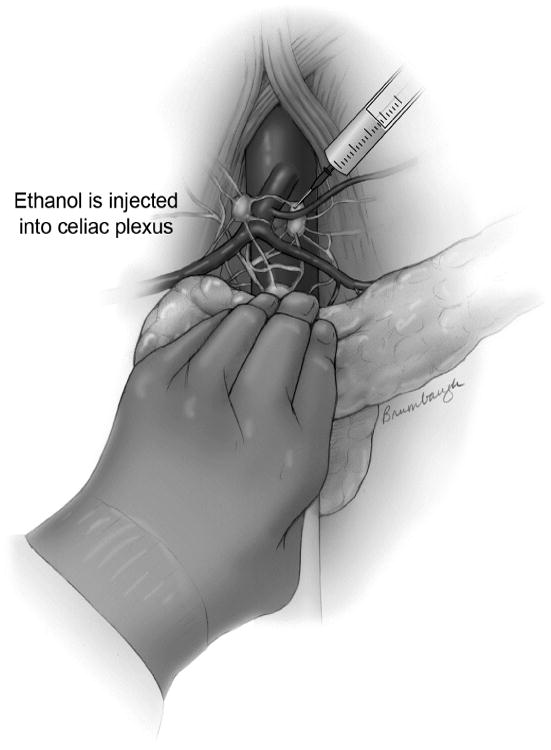
Intraoperative ethanol celiac plexus neurolysis procedure with injection of 50% ethanol into the celiac nerve plexus. A 20 mL volume injection was performed on each side of the aorta. (Reprinted courtesy of the artist, Jennifer Brumbaugh.)

Patients believed to harbor resectable PPA underwent exploratory laparotomy with confirmation of tumor resectability. If in fact the cancer was determined to be resectable (as preoperatively assessed), either pancreaticoduodenectomy or distal pancreatectomy with end-bloc splenectomy was performed depending upon the tumor's location. Patients identified at the time of exploration to have unresectable PPA underwent open biopsy (if indicated), and palliative biliary or gastrointestinal bypass as were deemed appropriate. Once the determination of resectability was made, patients were randomized to receive either the study drug or placebo control. They were stratified by tumor resectability (resectable/unresectable) and the presence or absence of preoperative pain (pain/no pain). ECPN was performed as a 20 ml injection of 50% ethanol in saline, delivered with a 20 gauge spinal needle on each side of the aorta at the level of the celiac axis (total volume = 40 ml). The control (placebo) arm received the same volume injection of normal saline (0.9%). (Figure 1) Both the operating surgeons and study subjects were blinded to the randomization arm. During the early postoperative period, pain control consisted of intravenous narcotic based patient-controlled analgesia and oral opioids as directed by the surgical staff. Epidural catheters were not used. Hospital morbidity and mortality rates as well as postoperative hospital length of stay were recorded. Following hospital discharge, patients were requested to complete follow-up questionnaires assessing their pain and QOL at three-month intervals until two-year survivorship was achieved or death occurred. Patients were mailed questionnaires at the appropriate time intervals. If the questionnaires were not returned within two weeks, the patient was called by trained study personnel. At that time, patients were reminded to complete the questionnaires or given the option to complete the questionnaires by phone. Demographic data (i.e., age, gender, and follow-up contact information) and clinical data (i.e., type of surgery, comorbid conditions, and postoperative complications) were obtained through outpatient and inpatient electronic medical records.

### Outcome Measures

The primary study endpoint of short and long-term pain was evaluated by the change in pain scores over time compared to the patients' preoperative baseline. Using numeric pain scores from the BPI survey (Likert scale from 0-10), the change in pain scores could be calculated to determine the effect of ECPN on tumor related pain. Secondary study endpoints including postoperative morbidity and mortality, QOL, and overall survival were also measured.

### Randomization

The TJU Division of Biostatistics conducted the patient randomization using computer generated random permuted blocks with a 1:1 allocation (blocks of six), with surgeons and staff blinded to the process. Patients were stratified by tumor resectability and the presence or absence of preoperative pain. For the purposes of baseline pain assessment, patients were considered to be in the pain stratum if they answered yes to the binary pain question, or if they noted a pain score of 3 or greater on the (0-10) Likert pain scale that is part of the BPI. The randomization and stratification resulted in four groups for comparison: resectable with pain (R/P), resectable with no pain (R/NP), unresectable with pain (UR/P), and unresectable with no pain (UR/NP). Following stratification, a central telephone number was called within the Department of Surgery where an administrator would open in sequence an envelope containing the assignment for celiac block injection. The assignment was then passed on to the Investigational Pharmacy to deliver the ethanol or saline injection to the operating room and the assignment sheet was delivered to the Division of Biostatistics to monitor the randomization process. A copy of the treatment assignment was maintained by the study coordinator and the Division of Biostatistics for monitoring and accrual tracking.

### Sample Size

The number of subjects needed for this study was based on the following assumptions for subjects with resectable tumors: Based on data reported in Lillemoe et al (1993), the proportion of patients experiencing pre-operative pain was estimated at 30%.^23^ Among those patients with preoperative pain, the expected proportion of patients in the control group with increased pain at 12 months was set at 50%. Among those patients without preoperative pain, the expected proportion of patients with increased pain at 12 months was set at 40%. To have 90% power to detect a common odds ratio of 0.25 using a two-sided Mantel-Haenszel adjusted chi-square test it was estimated that 58 subjects were required per treatment arm. An odds ratio of 0.25 is equivalent to a reduction in increased pain from 50% to 20% in those with preoperative pain and from 40% to 14% in those without preoperative pain.

Slightly different assumptions were made for subjects with unresectable tumors. The proportion of patients experiencing pre-operative pain was again estimated at 30%. Among those patients with preoperative pain, the expected proportion of patients in the control group with increased pain at 3 months was set at 90%. Among those patients without preoperative pain, the expected proportion of patients with increased pain at 3 months was set at 50%. To have 90% power to detect a common odds ratio of 0.25 using a two-sided Mantel-Haenszel adjusted chi-square test it was estimated that 57 subjects were required per treatment arm. An odds ratio of 0.25 is equivalent to a reduction in increased pain from 90% to 69% in those with preoperative pain and from 50% to 20% in those without preoperative pain.

To achieve 116 evaluable patients for the primary end point, we made the assumption that we would have a 60% survey return rate. As the study progressed, we found that our initial return rate was much lower than expected (20%), as was the number of unresectable patients that were being enrolled. Given these developments, with permission from our IRB, we increased the sample size to 170 patients with resectable tumors per arm and 72 patients with unresectable tumors per arm, for a total sample size of 485.

### Data Analysis

Continuous variables were summarized by stratum and randomization assignment using medians and inter-quartile ranges. Categorical variables were summarized using frequencies and percentages. Analyses were performed separately for resectable and unresectable patients. The primary outcome analyzed was a worsening of pain compared to preoperative baseline at 12 months in resectable patients or at 3 months in unresectable patients. In resectable patients, if the 12 month pain survey was not available, data were imputed from the 9 month survey response. If neither the 12-month or 9-month data were available then the 15 month survey was used. The relative risk of increased pain was calculated, adjusting for pre-operative pain strata using Mantel-Haenszel methods, and significance was assessed using the stratified-adjusted Mantel-Haenszel chi-square test. A secondary analysis of pain data in the subset of patients with resected PDA was performed, using mixed effects linear regression to model the longitudinal change in question 3 from the BPI (worst pain in the last 24 hours) during the first 12 months post-surgery. The survey month, treatment group and the interaction between these two terms were the main predictors included in both the multivariable and univariable models. Additionally the multivariable model controlled for diabetes, smoking history, tumor location (head or body/tail), age and baseline CA 19-9 levels. Models were fit separately based on whether a patient experienced pre-operative pain. Change in QOL was evaluated with a similar mixed effects model. Overall survival was summarized using Kaplan-Meier curves and groups were compared using log-rank tests. Treatment differences in incidence of complications were evaluated using Mantel-Haenszel chi-square tests. Differences in the number of complications were assessed using Poisson regression adjusted for stratum. The statistical software used was SAS v9.4 (SAS Institute Inc., Cary, NC). Significance was accepted at the p <0.05 level.

## Results

### Study Population

**Figure 2:**
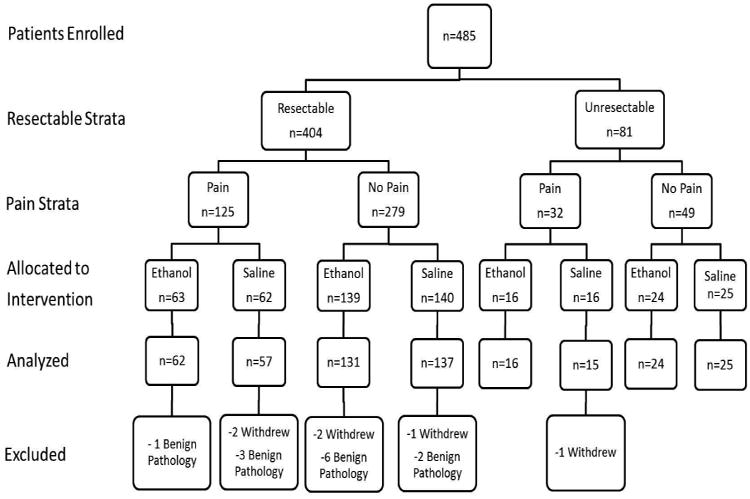
Ethanol celiac plexus neurolysis study design and flowchart.

From December 2008 to August 2013, 485 patients preoperatively suspected of having resectable PPA were explored, stratified, and randomized by preoperative pain status and tumor resectability for participation in this study (Figure 2). Of these, 12 patients were subsequently excluded from data analysis when they were found on final pathologic review to harbor benign disease, and 6 patients withdrew from the study after randomization, leaving a study cohort of 467 patients available for analysis. In total, 233 patients were randomized to receive ethanol and 234 patients to receive saline, and the patient allocation into sub-groups was 119 R/P, 268 R/NP, 31 UR/P, and 49 UR/NP.

**Figure 3:**
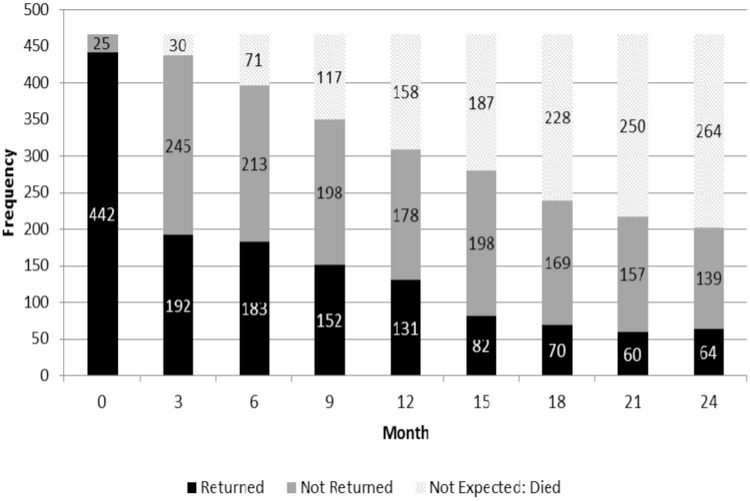
Survey response as a function of time.

Data on postoperative pain and QOL during the study period were collected and analyzed from the questionnaires mailed to study subjects. Figure 3 documents our response rate to the mailed surveys, incorporating patients who expired over the course of the study. Taking this factor into account, we averaged a 44% survey response over the first 12 months of the study, which was reduced to 30% in the second 12 months of the study. Unfortunately, our response rate was lower than we anticipated, and in many cases we were not able to capture complete data – a clear limitation of this study. Patient demographics and clinical variables for all study participants were comparable between the ethanol and saline groups as concerns, median age (68 v 67 years), female gender (46% v 42%), weight (74.8 v 75.6), and albumin (4 v 4), respectively (Table 1). There was a trend toward reduced rates of preoperative diabetes (25% vs 35%) and smoking history (34% vs 42%) in the ethanol group. Approximately 70% of patients had a primary pancreatic adenocarcinoma, and 91% of the resections were pancreaticoduodenectomies, while 9% were distal pancreatectomies. In keeping with CONSORT guidelines, we refrained from performing significance tests of baseline differences, though variables with apparent imbalances were incorporated into the multivariable regression analysis.^32^

The overall perioperative complication rate among only those patients undergoing resection was comparable between the ethanol and saline groups (44% vs 42%, p=0.75) (Table 2). The ethanol group showed a trend towards lower rates of pancreatic fistula (11% vs 17%, p=0.12) and intra-abdominal abscess (8% vs 11%, p=0.24), but a higher rate of delayed gastric emptying (DGE) (21% vs 17%, p=0.29), although none of these findings reached statistical significance. Combining both groups, the median length of stay for all resectable patients was 7 days, and the 30 day and 90 day mortality rates were 1.8% and 2.8%, respectively. Patients with unresectable tumors as a whole experienced a much lower rate of complications as would be expected, but between the ethanol and saline groups the rates were similar (14% vs 11%, p=0.39), respectively.

### Primary Outcome- Worsening of baseline pain

The primary outcome of the percentage of PPA patients experiencing a worsening of pain compared to their preoperative baseline was assessed at 12 months post-surgery for resectable patients. There was no difference seen in the primary outcome variable between the ethanol and the saline groups at the 12 month time point in either the R/P stratum (22% vs 18%, RR 1.23 (0.34, 4.46)), or the R/NP stratum (37% vs 34%, RR 1.10 (0.67, 1.81)), respectively (Table 3). When analyzing the primary endpoint as a function of time post-surgery, R/P ethanol patients appeared to show a trend toward reduced pain through 9 months with a subsequent rebound increase at 12 months, as compared to those who received saline (Table 4). However, neither the difference in pain scores nor the change from baseline as a function of time was found to be statistically significant. As a function of time, the R/NP group showed very similar pain scores between ethanol and saline groups, with both groups directionally demonstrating a gradual increase in pain from baseline over the follow-up period.

In the unresectable PPA groups (data not shown), there was no difference between ethanol and saline in the percentage of patients who experienced a worsening of pain compared to preoperative baseline at the pre-defined 3 month post-surgery time point: UR/P stratum (20% vs 0%, RR inf), and UR/NP stratum (14% vs 46%, RR 0.31 (0.05, 2.16), although the numbers of respondents in each cell became quite small. As a function of time, the UR/NP stratum had a significant reduction in pain in the ethanol group at 3 months compared to the baseline measurement (17% vs 44%, p=0.01), however this seemed to rebound to increased pain levels in the ethanol group by 6 months (50% vs 38%). Again, the numbers were quite small at each of these time point measurements.

**Figure 4:**
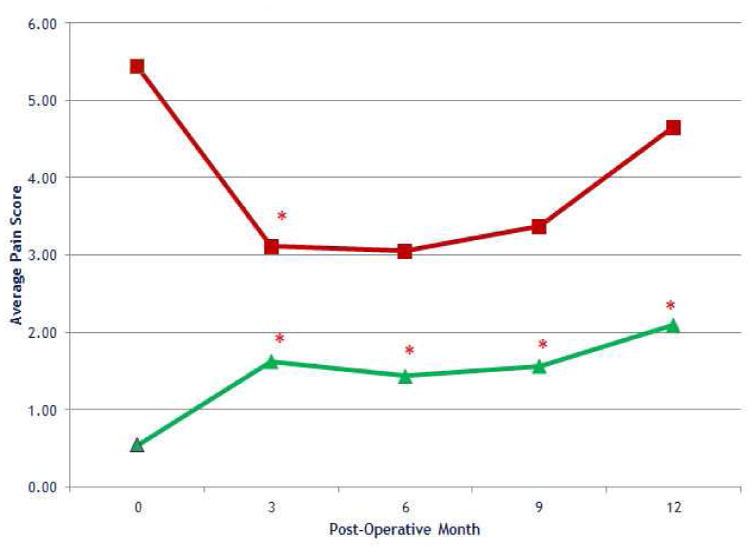
Average pain scores over time by preoperative pain in the subgroup of resectable, pancreatic ductal adenocarcinoma patients (using estimates from multivariate analysis) (*p<0.05). Red square, pain; green triangle, no pain.

Table 5 shows the results of a multivariable mixed effects model, looking at the average pain scores and change from preoperative baseline when the ethanol and saline groups were combined, for the subset of all resected patients with PDA. The model accounted for survey month, treatment group, age, diabetes, smoking history, tumor location, and baseline carbohydrate antigen (CA) 19-9 levels. There was a statistically significant reduction in pain at the 3 month time point following surgical resection in the R/P group, which maintained its magnitude in terms of absolute pain reduction to 9 months post-surgery, suggesting that surgical resection of the malignancy alone decrements pain levels to a significant degree among patients with preoperative pain (Figure 4). In the R/NP group, there is a small yet significant increase in pain at 3 months post-surgery (p=0.001), which remained fairly constant to 9 months post-surgery.

### Secondary Outcomes: QOL and Overall Survival

**Figure 5:**
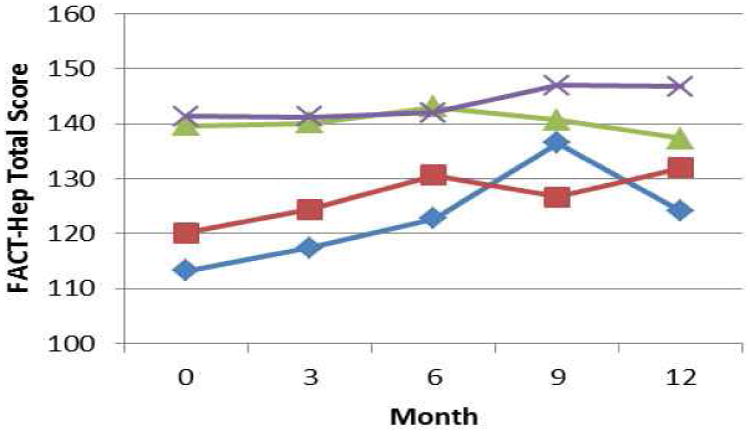
FACT-Hep total scores over time by pain and treatment group in resectable patients. Blue line, pain/ethanol; red line, pain/saline; green line, no pain/ethanol; purple line, no pain/saline.

QOL outcomes, as measured by FACT-Hep (version 4), demonstrated that patients with preoperative pain had a worse QOL compared to those without preoperative pain. Figure 5 graphically depicts the FACT-Hep total scores (higher score = improved QOL) for resected patients. The highest achievable score on this scale is 180, and patients with preoperative pain initially averaged lower QOL scores (116) than those without preoperative pain (141) (p<0.01), and this difference remained consistent throughout the study period. However, within each stratum, there were only minimal differences between the ethanol and saline groups. The same trend existed for unresectable patients, with the average FACT-Hep total score being lower in the UR/P group (119) as compared to the UR/NP group (134) (p=0.18).

**Figure 6:**
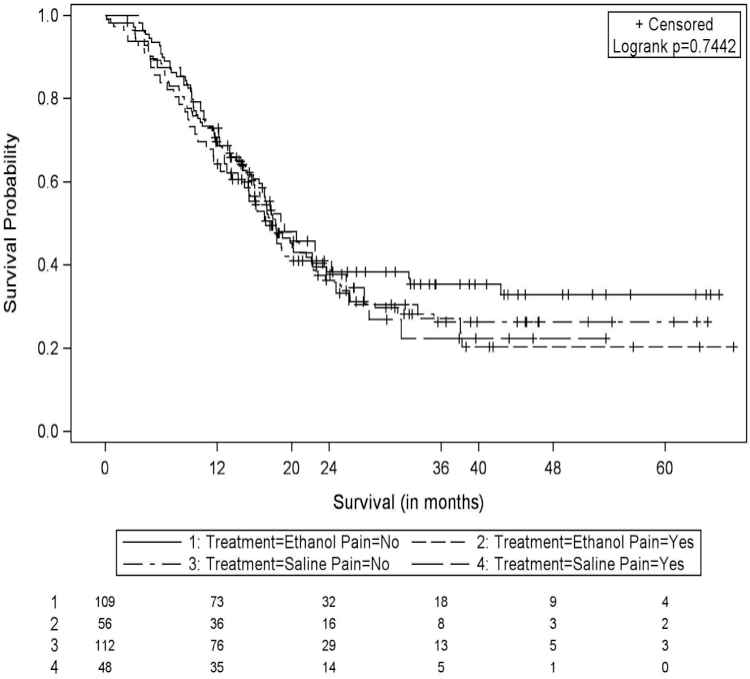
Kaplan-Meier survival estimates; resectable stratum.

When examining only the subset of patients with resected PDA, the overall median survival between the groups was nearly identical at 18.3 months in the ethanol group, and 17.6 months in the saline group (Figure 6). There were also no differences in overall median survival when resected patients with preoperative pain (18.8 months) were compared to those without preoperative pain (18.2 months). The 5-year survival rate for resected PDA was 26.8%. For unresectable patients, the median survival in the ethanol group was 8.1 months, and for the saline group was 8.6 months. The median survival of unresectable patients with preoperative pain (5.2 months) showed a trend toward reduced overall survival as compared to those patients without preoperative pain (10.2 months), however the numbers were too small in these groups to reach statistical significance (p=0.16). The 1-year survival rate for unresectable PDA was 37.5%.

## Discussion

We have carried out a single center, prospective, randomized, double blind placebo controlled trial to assess the efficacy of ECPN in reducing pain in patients with resected PPA. Our data show a statistically significant reduction in pain among patients who presented with preoperative pain (R/P) following surgical resection of their malignancy, which was durable to 9 months post-surgery. However, the addition of ECPN to resection did not contribute to a synergistic effect and appeared to offer no benefit. The secondary endpoints of perioperative complications, QOL, and overall survival were equivalent between groups. Overall, these results suggest that ECPN does not offer a benefit for PPA patients who are undergoing surgical resection of their malignancy.

PPA related pain often presents as severe, unremitting epigastric abdominal pain, though a number of different pain syndromes are common. It frequently results from direct tumor involvement of the adjacent celiac nerve plexus, which is comprised of the celiac ganglia and a dense network of nerve fibers. The celiac nerve plexus transmits visceral afferents from much of the upper abdomen, including the region encompassing and surrounding the pancreas.^33^ The source of PPA-associated pain is not well understood, though it is theorized to result in part from a variety of processes, such as neuropathy secondary to direct cancer cell invasion of the celiac plexus, infiltration of the intrapancreatic nerves, chronic pancreatic ductal obstruction, invasion of neighboring organs and also may be a byproduct of cancer therapies such as surgery or radiation. ^12,15,28,34^ By the time PPA progresses to stage 4 advanced metastatic disease, up to 90 percent of patients will experience pain as one of their symptoms.^13^ Currently, the most commonly used approach to pain management relies on the titration of systemic medications to appropriate doses in an algorithm described by the World Health Organization (WHO) analgesic ladder.^19,35^ By using sequentially stronger classes of analgesics, it is estimated that pharmacotherapy can successfully manage pain in nearly 80% of patients.^13,19,35^

While pharmacological therapy alone is often successful, opioid medications still provide inadequate pain relief in an estimated 20% of cancer patients.^19,35^ In addition, as opiate dosages increase, they often produce multiple adverse side effects, most commonly constipation, nausea and vomiting, anorexia, sedation, delirium, and urinary retention.^13,19^ Given this side effect profile, other methods of pain relief, such as nerve blocks, are utilized in many patients with and without cancer. Neurolytic blocks produce analgesia by destroying nerve structures responsible for pain transmission. Disruption of these peripheral neural pathways can be achieved in a variety of ways including surgery, cryotherapy, or the injection of damaging chemicals such as ethanol, phenol, and glycerin.^36,37^ The ethanol nerve block causes nerve damage by extracting lipid compounds and precipitating cell membrane proteins, inducing a chemical neurolysis which leads to nerve fiber demyelination and subsequent axonal degeneration. This nerve fiber destruction leads to an inhibition of the transmission of afferent pain signals to the central nervous system.^38-40^

A number of studies have evaluated the use of ECPN in patients with unresectable PPA, with varied results. In the study by Lillemoe et al, patients with unresectable PPA received intraoperative ECPN while undergoing exploratory laparotomy and palliative bypass. One hundred thirty seven patients were randomized to receive either ECPN via an intraoperative injection of 50% ethanol or 0.9% saline placebo, and were followed for 24 weeks to determine the effect of ECPN on long-term pain relief. In patients that received ECPN, pain relief was significantly improved at 2, 4, and 6 months of follow-up (p<0.05). Patients in the ECPN group had reduced opioid consumption, suggesting improved pain management and, in those without pain at diagnosis, the time until the new onset of pain was delayed. Additionally, in a small subset of patients with preoperative pain, survival also improved significantly (p<0.0001).

In the second largest study of ECPN to date, Wong et al examined the use of endoscopic ultrasound guided (EUS-guided) ECPN in 100 patients with unresectable tumors that experienced pain. Patients were stratified based on cancer stage and then randomized in a double blind fashion to receive an alcohol nerve block or systemic analgesic therapy (SAT) alone with a sham injection at the same site. After the first week, both groups experienced a reduction in pain scores, however the 53% reduction from baseline in the ECPN group was significantly larger than the 27% reduction observed in the SAT group (p=0.005). Pain intensity continued to decrease gradually and remained significantly lower in the ECPN group through 24 weeks of follow-up (p=0.01). In addition, significantly fewer patients receiving ECPN reported moderate to severe pain over the first 6 weeks of follow-up, demonstrating an improvement in those patients with the greatest amount of pain (40% vs 14%, P=0.005). However, other measures including quality of life, opioid consumption, physical and functional well-being, and survival did not differ between the groups.

Based upon the success of these prior studies in patients with unresectable PPA, we hypothesized that ECPN may be beneficial in patients with resectable PPA. The procedure is relatively fast, inexpensive, and is associated with few side effects, most commonly transient hypotension and rarely diarrhea.^24,28,31^ If shown to be beneficial, ECPN would represent a good alternative to systemic pain therapy alone, while adding minimal risk to the patient.

In contrast to previous findings in unresectable patients, our data did not demonstrate a significant improvement in pain scores in patients with resectable PPA, suggesting limited to no benefit from the ECPN procedure in this cohort of patients. On multivariable analysis of a subgroup of PDA patients, we did note a reduction in pain through the first 9 months following surgery in the R/P group. This finding of reduced pain was attributable to surgical resection of the malignant tumor, without any added benefit from the addition of ECPN. In fact, surgical resection may have inadvertently masked the effect of ECPN on PPA associated pain. Interestingly, despite this improvement in pain after surgical resection, there were no corresponding appreciable improvements in QOL. In general, patients with preoperative pain experienced a reduced QOL compared to those without pain, however, there were no differences in QOL with the addition of ECPN over saline placebo. In the literature, surgical resection has been shown to result in an initial reduction in patients' QOL; this generally recovers within 6 months to preoperative levels. In the long term, most patients undergoing PPA resection eventually experience an equivalent QOL when compared to both healthy controls and other patients undergoing abdominal surgery. Resected patients have been observed to experience a better QOL than their unresected counterparts. ^41-45^

Overall, we were unable to show that the benefits of ECPN previously described in unresectable patients could be translated to patients undergoing tumor resection. Survival was equivalent between the ethanol and saline groups, in the cohorts of patients with both resectable and unresectable disease. The theoretical advantage of ablating the celiac nerve plexus and the associated PPA tumor cells that had presumable invaded the plexus along with them, did not translate to any improvement in survival among ECPN patients, compared to those who received saline. This finding would generally coincide with that of the literature, where improvements in overall survival with the addition of ECPN have been rare.^6,23,26^

In addition to examining the potential therapeutic effects of ECPN in patients with resectable PPA, we also attempted to validate the prior success of ECPN with unresectable disease. However, as this was not the primary focus of our study, we did not have adequate power in the unresectable arm and therefore many of the findings do not reach statistical significance. The small number of unresectable patients combined with the low survey response rate led to a lack of meaningful findings for this subgroup.

It is important to note that there are a number of significant limitations to this study. First, it was a single center trial and although the study size was large there was a disappointingly low questionnaire return rate which was used to assess the primary outcome of postoperative pain. We acknowledge this most important study limitation. This is a not an uncommon problem in QOL studies in this patient population, where study surveys can represent a significant additional burden on patients who are in many cases dealing with issues of increasing disability, cancer recurrence, and death over the length of the study period. ^46^ Second, there was incomplete information obtained from the study subjects on the use of narcotic analgesics, therefore reductions in opioid usage, which may indicate some degree of pain relief, were not documented. Third, we did not have complete and accurate information on the administration of adjuvant therapies, such as chemotherapy and radiotherapy. These have been shown to impact patients' pain experience and effect narcotic consumption when used alongside ECPN.^6^ Fourth, the prolonged follow-up period of 2 years was perhaps too lengthy as most prior studies have shown a benefit of ECPN extending to only 3-6 months. ^30,47,48^ Fifth, patients were not stratified according to tumor location as determined by final pathologic analysis, which besides impacting potential surgical techniques (pancreaticoduodenectomy vs distal pancreatectomy), has been shown to correlate with staging and symptoms at diagnosis.^4^ However, tumor location was incorporated into the multivariable regression model. Finally, because the study was powered to determine the effects of ECPN in patients with resectable disease, it was not possible to demonstrate an improvement in pain control in the unresectable arms as has been suggested in prior studies.

## Conclusion

In this study, the world's largest randomized controlled trial evaluating ECPN in patients with operable PPA, we have demonstrated no benefit to patients in terms of postoperative pain and quality of life, and no effect on overall survival. Therefore, we cannot recommend the use of ECPN to mitigate cancer related pain in resectable PPA patients. However, further well designed studies in the unresectable cohort, with a high rate of complete follow-up, survey return, and meaningful QOL assessment may be warranted.

## Figures and Tables

**Table 1:** Pancreatic and Periampullary Adenocarcinoma Patient Demographic, Preoperative, and Pathologic Characteristics

Total (n=467)	Ethanol (n=233)	Saline (n=234)
Age, y (Q1, Q3)	68 (58, 76)	67 (61, 76)
Sex, female, n (%)	107 (46)	98 (42)
Diabetes, n (%)	57 (25%)	81 (35%)
Hemoglobin A1c, mmol/mol (Q1, Q3)	5.9 (5.3, 6.6)	6.1 (5.5, 7)
Smoking history, n (%)	78 (34%)	98 (42%)
Weight, kg (Q1, Q3)	74.8 (63, 87)	75.6 (64, 89)
Albumin, g/dL (Q1, Q3)	4 (3.6, 4.3)	4 (3.7, 4.3)
Pancreatic ductal adenocarcinoma pathology, n (%)	165 (71%)	160 (68%)

**Table 2:** Complications among Resected Pancreatic and Periampullary Adenocarcinoma Patients

Total (n=387)	Ethanol (n=193)	Saline (n=194)	p Value
Complications, any	85 (44%)	82 (42%)	0.75*
Complication type			
Pancreatic fistula	22 (11%)	33 (17%)	0.12*
DGE	41 (21%)	33 (17%)	0.29*
Intra-abdominal abscess	15 (8%)	22 (11%)	0.24*
Wound infection	12 (6%)	17 (9%)	0.33*
UTI	16 (8%)	13 (7%)	0.58*
Cardiac	12 (6%)	11 (6%)	0.83*
HJ or DJ leak	6 (3%)	9 (5%)	0.42*
Other	9 (5%)	8 (4%)	0.77*
No. of complications per patient, mean	0.7	0.8	0.28†
LOS, d (median, range; Q1, Q3)	7 (6, 10)	7 (6, 8)	
30-d Mortality, n (%)	4 (2.1)	3 (1.5)	0.66*
90-d Mortality, n (%)	4 (2.1)	7 (3.6)	0.37*

Fistula grading per ISGPF criteria.

* Cochran-Mantel-Haenszel: row mean scores difference p-value.

† Zero-Inflated Poisson: treatment group p-value.

DGE, delayed gastric emptying; UTI, urinary tract infection; DVT, deep venous thrombosis; HJ, hepaticojejunostomy; DJ, duodenojejunostomy; LOS, postoperative hospital length of stay.

**Table 3:** Primary Outcomes: Percentage of Pancreatic and Periampullary Adenocarcinoma Patients Who Experienced a Worsening of Pain Compared to Their Preoperative Baseline

Resectable/pain			Resectable/no pain		
Ethanol	Saline	Relative Risk	Ethanol	Saline	Relative Risk
5/23 (22%)	3/17 (18%)	1.23 (0.34,4.46)	19/51 (37%)	21/62 (34%)	1.10 (0.67,1.81)

Mantel-Haenszel relative risk: 1.12 (0.70, 1.78); p=0.64. Resectable stratum at 12 month time point.

**Table 4:** Resectable Stratum: Percentage of Pancreatic and Periampullary Adenocarcinoma Patients Who Experienced a Worsening of Pain Compared to Preoperative Baseline (Monthly Outcomes)

Month	Resectable/pain		Resectable/no pain	
	Ethanol	Saline	Ethanol	Saline
3	2/19 (11%)	3/16 (19%)	10/47 (21%)	15/52 (29%)
6	4/18 (22%)	3/16 (19%)	10/51 (20%)	15/51 (29%)
9	2/15 (13%)	4/13 (31%)	9/38 (24%)	12/44 (27%)
12	4/14 (29%)	0/10 (0%)	10/30 (33%)	17/44 (39%)
15	3/9 (33%)	2/8 (25%)	6/19 (32%)	13/29 (45%)
18	0/7 (0%)	1/9 (11%)	3/15 (20%)	12/24 (50%)
21	2/9 (22%)	1/6 (17%)	6/14 (43%)	3/17 (18%)
24	0/7 (0%)	2/7 (29%)	4/13 (31%)	5/19 (26%)

**Table 5:** Multivariate Analysis: Average Pain Scores and Change From Baseline in the Subgroup of Resectable, Pancreatic Ductal Adenocarcinoma Patients

	Pain			No Pain		
Month	Pain Score	Change from Baseline	p Value	Pain Score	Change from Baseline	p Value
0	5.43	--	--	0.54	--	--
3	3.11	-2.32	0.036	1.62	1.08	0.001
6	3.05	-2.38	0.075	1.43	0.89	0.022
9	3.37	-2.06	0.067	1.56	1.02	0.014
12	4.65	-0.78	0.512	2.09	1.55	0.001

Multivariate model adjusts for survey month, treatment group, age, diabetes, smoking history, tumor location, and baseline carbohydrate antigen (CA) 19-9 levels.
